# Supplementary material for: Differences between 3D isovoxel fat suppression VIBE MRI and CT models of proximal femur osseous anatomy: A preliminary study for bone tumor resection planning
Source: PLoS One. 2021 Apr 30;16(4):e0250334. doi: 10.1371/journal.pone.0250334 (PMC8087022; doi:10.1371/journal.pone.0250334)
Supplement: Attachment — Submitted filename: Response to reviewers.docx [file pone.0250334.s003.docx]

**Response to reviewers**

Reviewer #1-1: Abbreviations, such as VIBE, should be explained at the first time it appears in your article. You can put it in your title, but for both abstract and your manuscript, you should give the full text.

Answer: Thank you for your comment. We have given the abbreviation at the first appearance of the abstract and manuscript.

Reviewer #1-2. But Dixon and WE based fat suppression techniques are not based on the saturation of the fat. It is not proper to call them fat saturation techniques.

Answer: Thank you for your comment. We modified the term ‘fat saturation’ into ‘fat suppression’ throughout the manuscript.

Reviewer #1-3. Figures are quite low quality for the authors to see clearly. For example, the figure 2, the text in sub-figures can not be seen clearly at all.

Answer: The figure 2, and 3 are modified and reformatted. We have omitted some unnecessary sub-figures, which seems to be not informative.

Review #1-4. The authors should give explanation for why fat suppression is necessary for the recon of the bone. Also the 3D osseous anatomy of the proximal femur extracted without fat suppression should be given as a baseline for comparison.

Answer: Thank you for your suggestion. We added the explanation for the reason why fat suppression was necessary in discussion section. “Suppression of the bone marrow was necessary to avoid chemical artifacts, which may be problematic especially in the femoral head area. The thickness of subchondral bone plate of femoral head measures below 0.9mm, which would be smaller than the size of a voxel.” This chemical artifact was noticed during the pilot study, and we concluded that fat suppression would be necessary when planning the study design.

Review #1-5. Dixon based fat suppression are totally different from the WE in principle. So, it is quite interesting to figure out why they are equal in the discussion, from the medical physics view. It is expected that the authors would give more evidences, such as other reports.

Answer: Referring to the previous report of Kirchgesner et al that Dixon method was more effective in fat suppression and yielded higher signal to noise ratio compared to chemical shift-selective technique for 3D T1-weighted MR imaging, the authors chose WE method as a comparative arm for Dixon fat suppression method. There were no previous studies were found in the literature comparing these two fat suppression methods.

Review #1-6. References 14 and 15, seems some typos of the authors' name

Answer: Thank you for your comment. We have corrected the author’s name in the references.
